# Incorporation of PVDF Nanofibre Multilayers into Functional Structure for Filtration Applications

**DOI:** 10.3390/nano8100771

**Published:** 2018-09-29

**Authors:** Remi Roche, Fatma Yalcinkaya

**Affiliations:** 1 National Polytechnic Institute of Chemical Engineering and Technology (INP-ENSIACET), 4, Allée Emile Monso-CS 44362, 31030 Toulouse CEDEX 4, France; remiroche71@sfr.fr; 2 Department of Nanotechnology and Informatics, Institute of Nanomaterials, Advanced Technologies and Innovation, Technical University of Liberec, Studentska 1402/2, 46117 Liberec, Czech Republic; 3 Institute for New Technologies and Applied Informatics, Faculty of Mechatronics, Studentska 1402/2, 46117 Liberec, Czech Republic

**Keywords:** electrospinning, PVDF, HEPA filter, wastewater, nanofiber

## Abstract

Membranes are considered as a promising technology for separation and filtration processes. Here, novel polyvinylidene fluoride (PVDF) nanofibrous multilayer membranes were fabricated by wire-based industrial electrospinning equipment following by a lamination process. The lamination process was optimised under various applied temperature, force of lamination, and lamination time. Air permeability and burst-pressure tests were run to determine the optimum membranes for filtration application. The structures of the prepared membranes were characterised by scanning electron microscopy and pore-size analysis. The hydrophilic properties of the membranes were evaluated using water contact angle measurement, and the mechanical strength of the membranes was analysed. Air and water filtration tests were run to find the possible application of prepared membranes. The air filtration results showed that membranes had high filtration efficiencies: Over 99.00% for PM_2.5_, and PM_0.1_. The water filtration results indicated that permeability of the membranes changed from 288 to 3275 L/m^2^hbar. The successful preparation of such an interesting material may provide a new approach for the design and development of electrospun filter membranes.

## 1. Introduction

Air and water pollution has become a major environmental concern due to the growing global population, increasing economic expansion, and amount of consumption. Many developing countries face an increase in particulate matter (PM) as an air pollutant and amounts wastewater [1,2,3,4]. One of the greatest challenges to the sustainability of modern society is an inadequate supply of clean air and water. Due to its easy operating conditions, no demand for toxic chemicals, energy saving, and cost-effective features; membrane technology has become an emerging and indispensable technology for air filtration and water purification.

The versatility of nanotechnology could greatly help to overcome the current issues of cleaning of air and water using novel nanostructured membranes produced by the electrospinning process. Electrospinning, a simple and cost-effective method, has been used extensively to produce nanofibres with high pore volumes, small pore size, fibre diameters of nano size with tightly controlled size distribution, and high surface area to volume ratio. Nanofibrous membranes are made of randomly laid fibres oriented anisotropically that can effectively filter out particles by a size-exclusion mechanism. Electrospun polyvinylidene fluoride (PVDF) nanofibrous membranes find applications in many areas such as membrane distillation [5], microfilters [6,7], air filters [8], electrically conductive nanofibres [9,10], and separators [11]. However, the poor mechanical properties of the nanofibres restrict their application in liquid filtration [12,13]. To overcome this problem, various attempts have been made, such as blending of the polymers [14,15,16,17], using epoxy composites [18], reinforced with nanomaterials [19,20,21], dip-coating [22], self-reinforcing methods [23], thermal lamination [24,25], etc. Silicon carbide (SiC) nanofibres were reinforced with an epoxy matrix cured with an anhydride hardener. Results showed a good level of adhesion between nanofibre and matrix in composites with lower nanofibre loading [26]. A nylon-6 nanofibre layer was fabricated and dip-coated in silicone film. Results indicated that the tensile strength of the fibres increased 63% compared to neat nylon-6 nanofibre alone [27]. These methods are generally costly, require toxic chemicals, are time consuming, and in some cases show low efficiency. Recently, a three-dimensional (3D) interconnected network was fabricated using a carbon nanofibre layer. The 3D interconnected fibre networks exhibited excellent stability and methanol tolerance to commercial Pt/C catalysts [28]. In another work [29], binder-free three hybrid-structured silicon-based anodes were developed from nanostructure and graphene sheets. The silicon (Si) nanoparticles were dispersed in a carbon precursor and polyvinyl pyrrolidone stabiliser for electrospinning. The Si/polymer nanofibres were dispersed in a graphite oxide solution followed by a vacuum filtration and thermal treatment. The resultant hybrid material was used as an electrode with no binder. Compared to thermal lamination, the 3D interconnected network method requires expensive compounds.

Here, we present a novel lamination method to use nanofibres in various applications. The aim of this work was to conduct the nanofibres into a filtration application using lamination technology and to measure their performance for both air and water filtration. Firstly, various lamination conditions were tested in terms of their effect on the mechanical and surface morphology properties of the nanofibres. Lamination of the fibre surfaces could significantly enhance the mechanical performance of the nanofibrous membrane. Later, the prepared membranes were used as filters and their performance was measured.

Furthermore, characterisation and evaluation of the nanofibrous membranes were carried out to relate their structural properties to their separation performance and properties. To the best of our knowledge, no such strategy has been reported on the preparation and characterisation of various laminated PVDF nanofibre membranes for both air and water filtration applications.

## 2. Materials and Methods

### 2.1. Preparation of Nanofibre Layers

**Figure 1:**
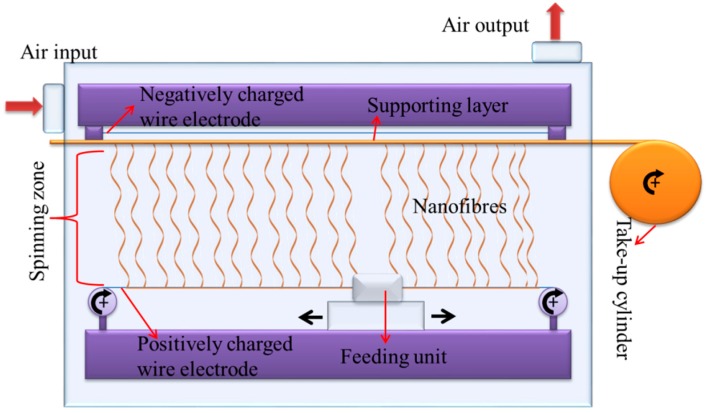
Schematic diagram of the needle-free electrospinning device.

PVDF (13 wt %, Solef 1015, Bruxelles, Belgium) was dissolved in *N*,*N*-dimethylformamide (DMF) (Penta s.r.o., Prague, Czech Republic) and mixed overnight. The prepared solution was electrospun using production-line needle-free electrospinning equipment (Nanospider NS 8S1600U, Nanovia, Czech Republic). In this technology, two types of wire electrode are placed upward (connected to a negatively charged high voltage supply) and downward (connected to a positively charged high voltage supply). The downward wire, called the feeding wire, is placed from one side to the other of the equipment in a horizontal direction and passes along the feeding unit, as shown in Figure 1. The feeding unit is connected via pumps to the solution tank filled with a polymeric solution. As the feeding unit moves backwards and forwards on the wire, the positively charged wire is wetted. If the electrical field overcomes the surface tension of the solution, fibres form on the surface of the downward wire and move towards the upward wire. An antistatic mobile supporting layer (such as backing paper or nonwoven web) is placed between the two wires. The nanofibres formed are collected onto the supporting layer. The resultant fibres are wound on a cylinder via a take-up cylinder. Humidity and temperature were controlled.

In our work, the downward wire electrode was maintained at +55 kV while the upward electrode was maintained at −15 kV. The distance between the electrodes was 188 mm. The distance between the upward electrode and the supporting backing material was 2 mm. The speed of the backing material was 20 mm/min. The temperature and humidity of the input air were set to 23 °C and 20% by the air-conditioning system. The nanofibre layer was collected on a backing paper at 3 g/m^2^.

### 2.2. Preparation of Nanofibrous Membranes

**Figure 2:**
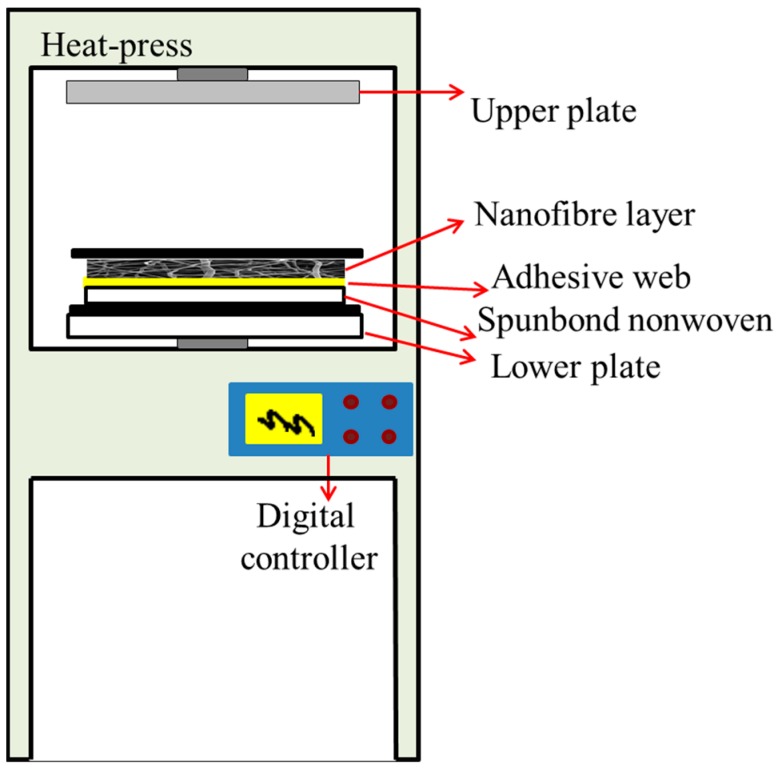
Schematic design of the heat-press equipment and replacement of the multilayer nanofibrous membranes.

The nanofibrous membranes were composed of a 3 g/m^2^ PVDF nanofibre layer, a 12 g/m^2^ co-polyamide adhesive layer (Protechnic, Cernay, France) and a 100 g/m^2^ polyethylene terephthalate spunbond nonwoven layer (Mogul Co., Ltd., Gaziantep, Turkey). Firstly, the prepared PVDF nanofibres were cut into A4 size and then placed on the adhesive web. The nonwoven supporting layer was placed on the other side of the adhesive web to create a nanofibre-adhesive web-nonwoven sandwich structure. Heat-press equipment (Pracovni Stroje, Teplice, Czech Republic) was used for the lamination process (Figure 2). In this equipment, there are two metallic hot plates (upper and lower). The sandwich structure was placed between the two hot plates. Different nanofibrous membranes were prepared using various lamination force, temperature, and lamination time. The conditions of the lamination and sample abbreviations are given in Appendix A Table A1. The abbreviation of the samples are given according to polymer temperature time force of lamination.

Hence there were 36 different samples; the most promising samples were selected for further measurements according to their air permeability and resistance to delamination. Higher air permeability and burst pressure are better for applications, due to the high mechanical demands placed.

### 2.3. Mechanical Properties of the Membranes

Tensile tests of the selected nanofibrous membranes were performed using a universal testing machine (Labor-Tech s.r.o., Opava, Czech Republic) with an extension rate of 10 mm/min at room temperature in dry conditions. The samples were 100 mm long, 25 mm wide, and the distance between the two clamps was 50 mm. Both machine direction (MD), and the counter direction (CD) were tested. At least three measurements were taken for each sample.

**Figure 3:**
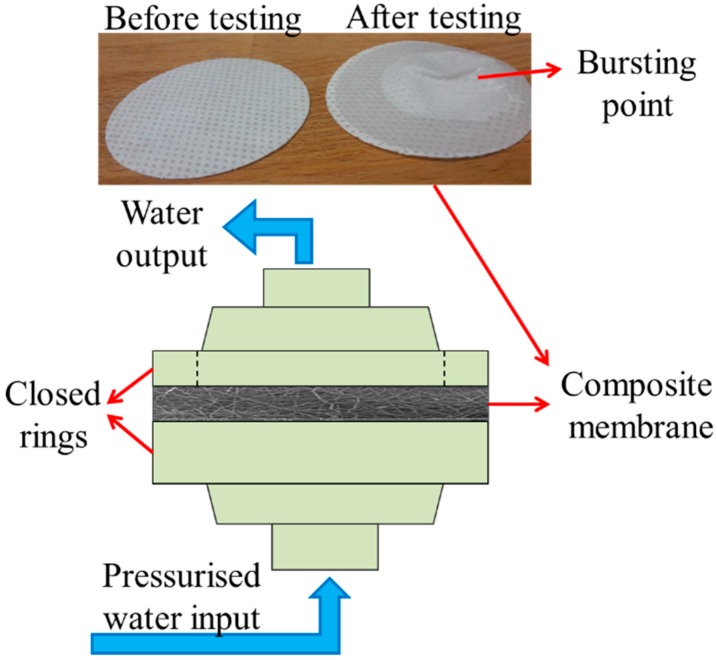
Burst-pressure testing unit.

The delamination of the nanofibrous membrane was tested, and the maximum burst pressure was recorded. The testing device was developed in our laboratory, and is shown in Figure 3. The principle of the device has been explained elsewhere [30]. In summary, the samples were placed between two rings, and the nanofibre side of the samples was placed on the upper side. Pressurised water was applied to the membrane, and the hydrostatic pressure was measured using a pressure controller from the face side of the membrane. The hydrostatic pressure was gradually increased, and as soon as the nanofibre layer burst, the pressure value on the screen decreased sharply. The maximum pressure value was recorded as the burst pressure of the membrane. At least three measurements were taken for each membrane. The nanofibre surface of the membrane was placed upstream.

The maximum, average and the minimum pore sizes are determined by a bubble point measurement device (developed in our laboratory), which was working with capillary flow porometry theory. Bubble point method has been explained in detail [31,32,33]. The bubble point test allows the size of the pores of the porous material to be measured. The pore flow means a set of continuous hole channels connecting the opposite sides of the porous material. At least three measurements were taken. In bubble point test, sufficient gas pressure is applied to overcome the capillary forces of the wetted membrane pores to determine largest pore size. The bubble point is the first stream of bubbles emerges at the largest pore.

### 2.4. Characterisation of Nanofibrous Multilayer Membranes

Surface morphology of the membranes was examined using a scanning electron microscope (Vega 3SB, Brno, Czech Republic) after the lamination process. At least 50 fibres were measured for each sample. Image-J (free online software) was used to determine fibre diameters.

The water contact angle of the samples was measured using a Krüss Drop Shape Analyzer DS4 (Krüss GmbH, Hamburg, Germany), at five different points, using deionised water (surface tension 72.0 mN/m, deionised by Aqual 27, Brno, Czech Republic) on the clean and dry samples at room temperature.

The maximum, average and the minimum pore sizes were determined by a bubble-point measurement device working with capillary-flow porometry theory, and which was developed in our laboratory.

The air permeability of all multilayer nanofibrous membranes was tested using an SDL ATLAS Air Permeability Tester (@200 Pa and 20 cm^2^, South Carolina, USA). At least three measurements were taken for each sample. The nanofibre surface of the membrane was placed upstream.

### 2.5. Filtration Test

The particle filtration test for the selected membranes was by an MPF 1000 HEPA filtration device (PALAS GmbH, Karlsruhe, Germany) for air filtration. The filtration efficiency for PM_2.5_ and PM_0.1_ was measured.

**Figure 4:**
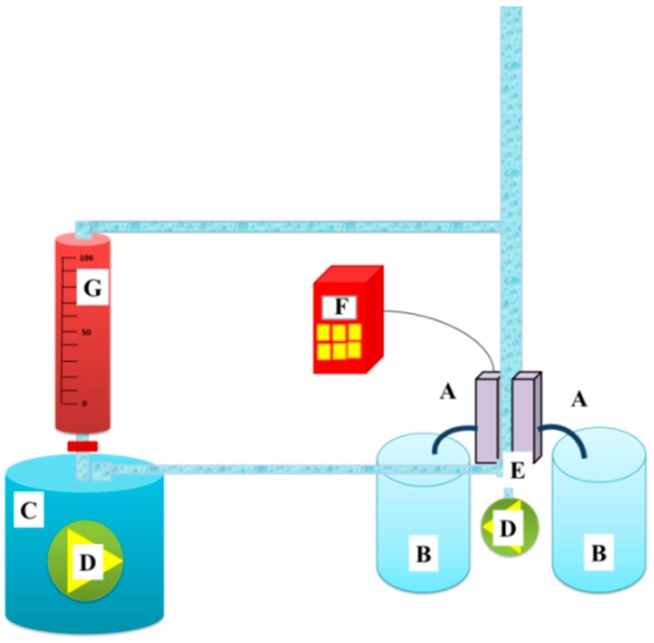
A cross-flow unit: (**A**) membrane cells, (**B**) permeate, (**C**) feed, (**D**) pump, (**E**) surface bubble cleaning, (**F**) pressure controller, and (**G**) feed flow speed controller.

A cross-flow filtration unit developed in our laboratory, as shown in Figure 4, was used for water filtration. 1500 mL of tap water was used as the feed. The flux (F) and the permeability (k) of the selected membranes were calculated according to Equations (1) and (2):

(1) $$F=\frac{1}{A}\frac{\mathrm{dV}}{\mathrm{dt}}$$

(2) $$k=\frac{F}{p}$$

where A is the effective membrane area (m^2^), t is the filtration time (h), V is the total volume of the permeate (L), p is the transmembrane pressure (bar), and t is the filtration time. The nanofibre surface of the membrane was placed upstream.

In water filtration test, the nanofibre surface of the membranes was in contact with feed solution.

## 3. Results and Discussion

### 3.1. Determination of Selective Membranes

**Figure 5:**
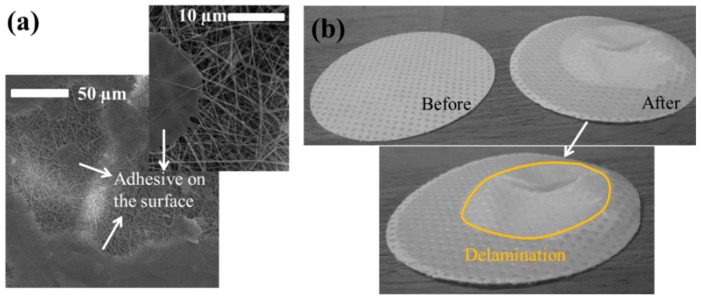
(**a**) Adhesive cover the surface of nanofibre, and (**b**) membranes before and after the burst-pressure test.

The mechanical strength of a single nanofibre layer is not suitable for application in air or water filtration. The main reason to laminate the nanofibre layer is to improve its strength against tearing or bursting, using a supporting layer. The lamination process is quite challenging. An improper lamination can cause burning of the nanofibre layer, blocking of the pores with adhesive or easy delamination from the surface of the supporting material. To determine the best lamination condition, various system parameters were changed, such as applied temperature, force of lamination or time of lamination. Laminated samples were subjected to air permeability and burst-pressure tests to select the most suitable membranes for further applications. The main reason for selecting these two tests can only be explained as: (a) The air permeability test shows whether all the pores or almost all pores of the nanofibre layer are blocked with adhesive. Membranes with the highest permeability have advantages in air and water filtration, and (b) burst-pressure tests indicate the maximum pressure that the membrane can withstand without delaminating and separating the nanofibres from the supporting layer. Figure 5a shows the adhesive web covering the surface of the nanofibre and blocking the pores. Figure 5b shows the membrane before and after the burst-pressure test. The bursting nanofibre layer forms a cone shape on the supporting layer.

The air permeability and the maximum burst pressure of the membranes are given in Table A2.

The density of the nanofibrous membranes can be one of the biggest problems in the air permeability test. The high density means a thicker sample, which reduces the air flow.

**Figure 6:**
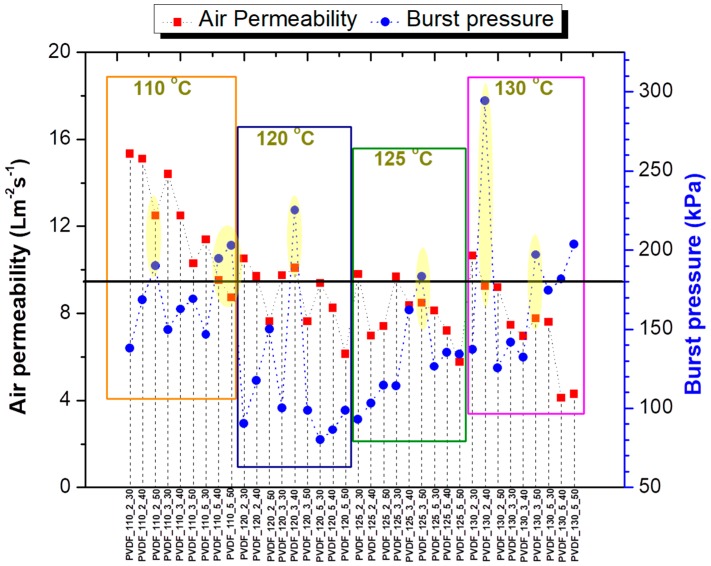
Determination of membranes for filtration application.

Hence, almost all the samples showed very low air permeability; their burst pressure was considered as the criterion for the selection of membranes. Since there is no previous work dealing with the effect of lamination on membrane delamination and bursting, the degree of acceptable air permeability and the burst pressure was determined by us, based on previous experiences in our laboratory. From each temperature series, at least one membrane was selected. In this case, the membranes with burst pressures over 175 kPa and air permeability ≥7 Lm^−2^s^−1^ were selected as promising candidates for filtration application. Only seven membranes showed reasonable air permeability with high burst pressure. The results of air permeability and burst pressure of all samples are given in Figure 6. Graphics are divided into four regions due to applied lamination temperature. In each region, each point indicates the membranes with the same abbreviation and sequence order as in Table A2. For instance, the first point in each region indicates a membrane laminated for 2 min at 30 kN, while the last point indicates five min at 50 kN. The samples laminated below 100 kN were not considered due to very low air permeability. A black line was drawn at 175 kPa to determine the minimum limit for the burst pressure. The selected membranes are indicated in Table A2 (*) and in yellow regions in Figure 6.

Results indicated that applied force of lamination is the most effective lamination parameter compared to temperature and lamination time. Based on the results, one can understand that a force over 100 kN or less than 40 kN was not suitable for the lamination process of PVDF nanofibres. When the applied force was lower (i.e., 30 kN) membranes delaminated and burst easily. Most of the selected samples were prepared below 110 °C, which leads to energy saving for real applications.

### 3.2. Tensile Strength

Tensile testing is one of the most efficient methods to measure tensile properties of nanofibrous membranes. Using a single nanofibre layer, it is not possible to measure the tensile strength due to the low mechanical properties of nanofibres. For this reason, the tensile property of the nanofibrous membranes depends on the tensile properties of the supporting layer used for lamination of the nanofibre layers. The tensile strength of the membranes shows the resistance of the membranes to tearing under pressure or load. Table 1 shows the tensile strength (N/25 mm) and the elongation at break for some selected materials. Both machine (MD) and counter direction (CD) of the membranes were tested. The shape changes of the membranes before cracking was shown by elongation at break.

The results indicate that the nonwoven supporting layer had an anisotropic structure, which resulted in different tensile strength and elongation at break for the MD and CD of the membranes. The lamination parameters did not show an important effect on the tensile properties. The results indicate that the membranes had a very high tensile strength that could withstand external forces or pressures.

### 3.3. Surface Characterisation

**Figure 7:**
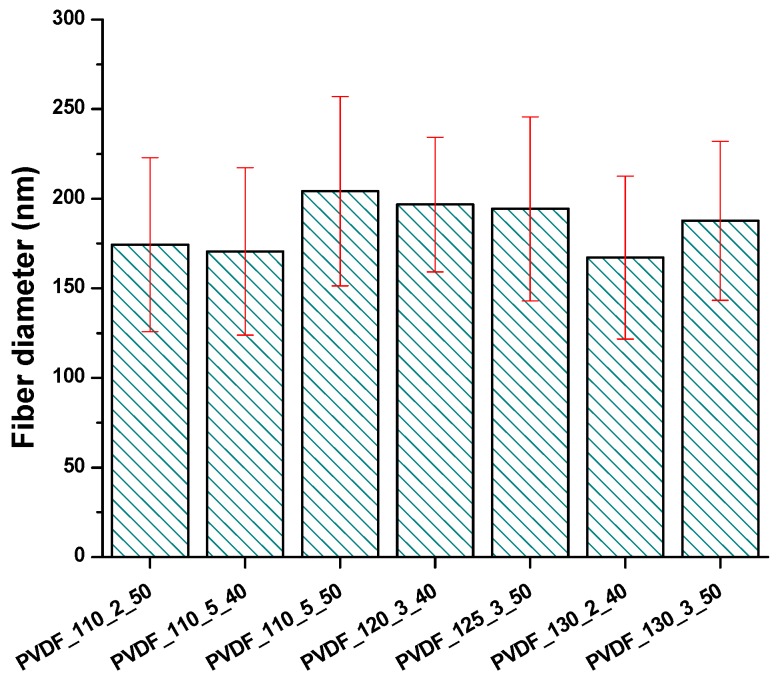
The fibre diameter of various nanofibrous membranes.

**Figure A1:**
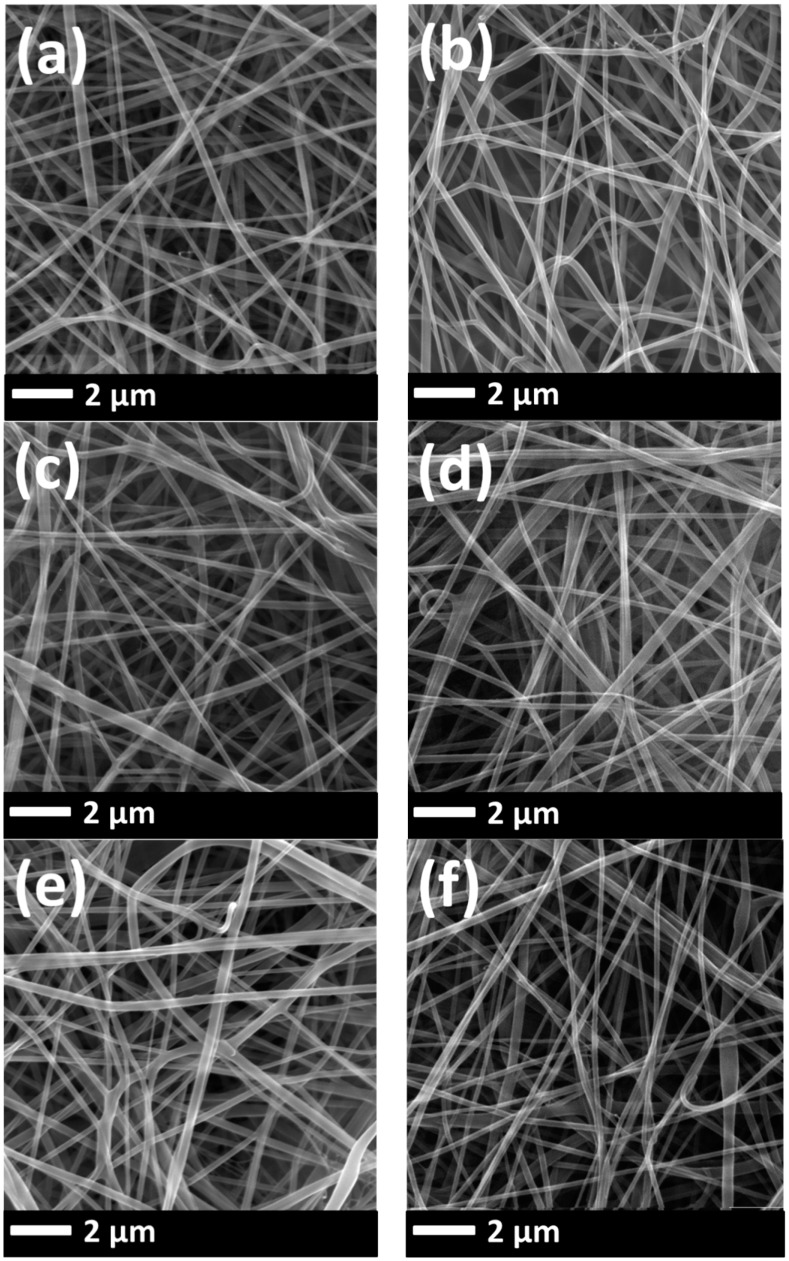

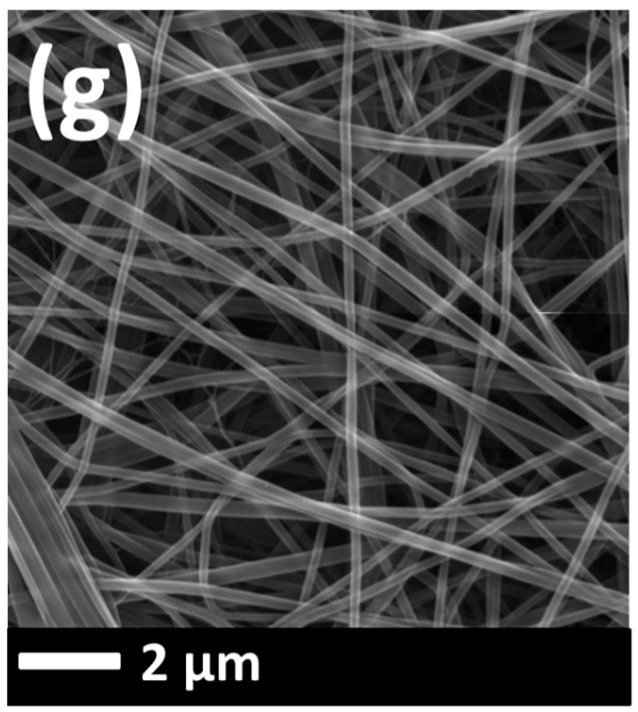
SEM images of (**a**) PVDF_110_2_50, (**b**) PVDF_110_5_40, (**c**) PVDF_110_5_50, (**d**) PVDF_120_3_40, (**e**) PVDF_125_3_50, (**f**) PVDF_130_2_40, (**g**) PVDF_130_3_50 after lamination process.

The surface morphology of the nanofibrous membranes was investigated using SEM images (Figure A1). The fibre diameter was evaluated after the lamination process, as shown in Figure 7. Hence the membranes can be used only in laminated form; the diameter of the neat nanofibre web itself was not measured.

According to Figure 7, there were no important changes in the fibre diameter between various lamination conditions.

**Figure 8:**
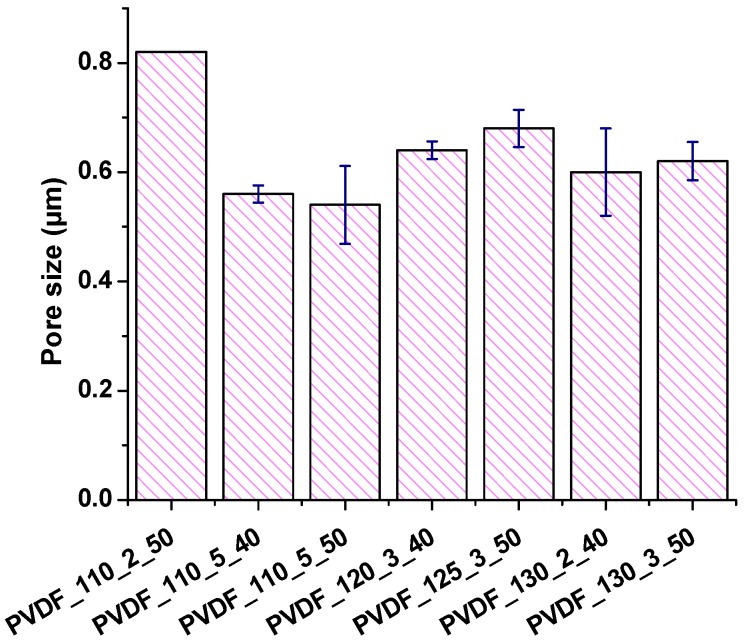
The average pore size of various nanofibrous membranes.

The average pore size of the selected membranes was measured and shown in Figure 8.

The measurement of pore size showed that:

(1) Membranes which were laminated under the same temperature and force of lamination but different lamination duration indicated that higher lamination duration decreased the pore size.
(2) Membranes which were laminated under the same temperature and duration but different force of lamination showed that the pore size of the membrane did not change significantly.
(3) Membranes which were laminated under the same force of lamination and duration but different temperature showed that the pore size of the membrane slightly decreased with increasing applied temperature.
(4) All the membranes showed pore size below one µm.

**Figure 9:**
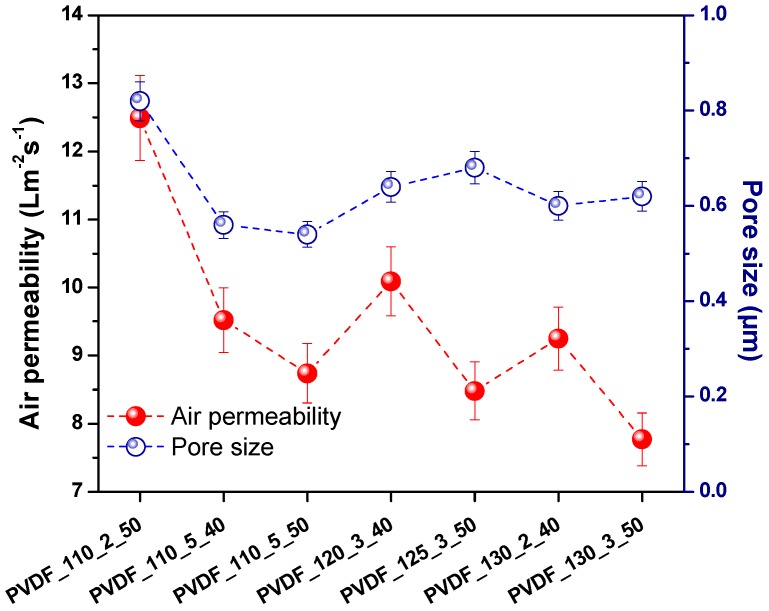
Pore size vs. air permeability of the nanofibrous membranes.

Figure 9 shows the dependence of pore size and air permeability. Almost all samples showed that air permeability changed in direct proportion to pore size. Surely, one should consider the non-porous areas existing on the membrane, which were the results of melting of the adhesive web. The non-porous area plays the biggest role in air permeability of the membranes. Figure 9 showed that the sample with the highest pore size had the highest air permeability.

**Figure 10:**
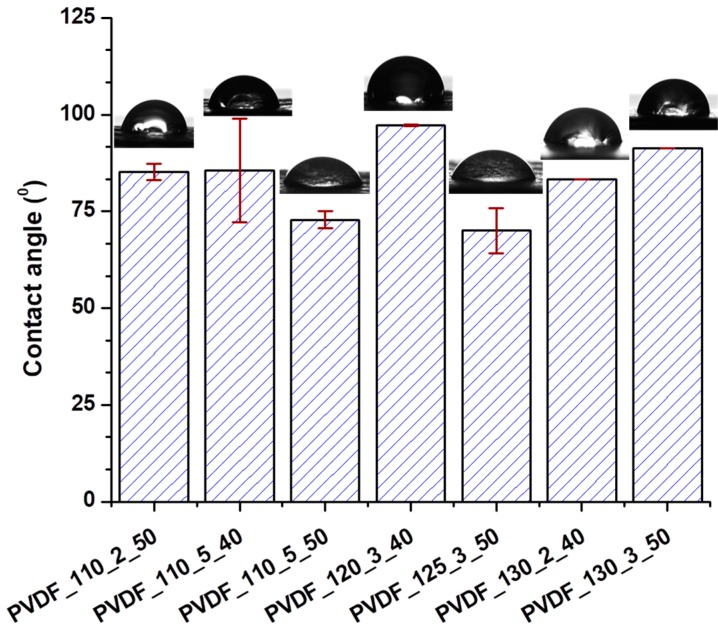
The contact angle of various membranes.

Water affinity is one of the important criteria for the permeability and wettability of the membranes for water domain filtration. It was found that both hydrophobic and super hydrophilic membranes were disadvantageous for use in the separation of wastewater [34]. The water contact angle of the membranes was measured as shown in Figure 10.

Based on the results of the contact angle, the lamination process showed the large effect on the hydrophilicity of the membranes. The neat nanofibre with no lamination showed a contact angle of around 115°. After lamination with a co-polyamide adhesive web, the wettability of the total membrane changed. It was found that the heat-press treatment slightly decreased the contact angle of the electrospun polyvinylidene fluoride-co-hexafluoropropylene membrane due to changes in the physical structure [35]. Our results showed that the lamination process played a large role in the wettability of nanofibrous membranes. The reason was that of the adhesive web that was covered on the surface of nanofibre layers partly.

### 3.4. Filtration Test

The selected membranes from Table A2 were examined for both air and water filtration. The aim of the air and water filtration tests was to determine the applicability of the nanofibrous membranes laminated under various conditions.

#### 3.4.1. Air Filtration

**Figure 11:**
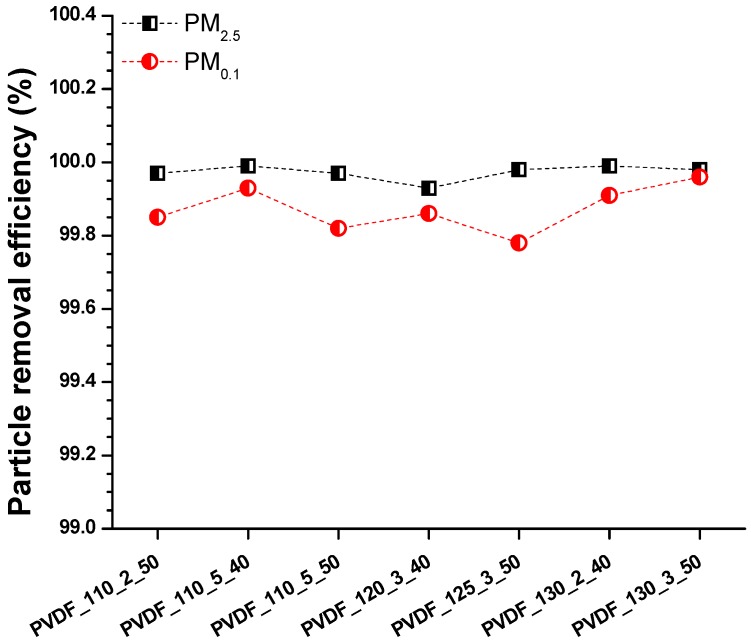
Particle removal efficiency for the various laminated nanofibrous membranes according to PM_2.5_ and PM_0.1_.

Nanofibre membranes are suitable for air filtration, catching fine particles due to the narrow pore size of the nanofibres. Using a neat nanofibre web, it is possible to achieve high filtration efficiency with low air permeability that is demanded. However, a nanofibre web by itself is soft and fragile and cannot be used as an air filter due to its poor mechanical properties. At least a second layer is required to support the nanofibre web to improve its mechanical weakness. Herein, a high-density PET spun bond was used as supporting material. The lamination process, with a high-density supporting layer and the high-density nanofibre web (3 g/m^2^), caused a decrease in the permeability of the total membrane, while increasing the filtration efficiency. In the literature, it was found that using a high density of nanofibre web and a multilayer system, the filtration efficiency of the material increased while air permeability decreased [36,37,38,39]. The air filtration efficiencies of the selected membranes (Table A2), according to PM_2.5_ and PM_0.1_, are given in Figure 11.

The physical structure of the nanofibres is one of the most effective parameters for the efficiency of air filters. It has been found that increasing the fibre diameter decreased filtration efficiency [40]. In another work, the surface area of the nanofibre web increased with lower fibre diameter, which led to a lower pressure drop [41]. In this work, the same nanofibre web was used under various lamination processes. Figure 7 shows that the fibre diameter for each sample did not show significant changes under various lamination conditions. Results indicated that all nanofibre layers had very good particle filtration according to PM_2.5_ and PM_0.1_, but with very low air permeability. Based on the results, it can be concluded that PVDF nanofibrous membranes, prepared by the heat-press method with a high-density adhesive web, supporting layer and nanofibre web are not good candidates for commonly used particle filters from the point of view of cost. Hence, the membranes had efficiency over 99% according to PM_2.5_ and PM_0.1_; those types of membranes could be used for special filters that require only high-efficiency filters against small particle size.

The fine particles around 2.5 µm can pass through the lungs into the blood and increase the risk of lung cancer. The particle diameter less than 2.5 µm, called ultrafine particles, are highly toxic to the lungs. The smaller particles are more harmful and can cause serious health issues. The membranes showed very good filtration efficiency for ultrafine particles.

#### 3.4.2. Water Filtration

**Figure 12:**
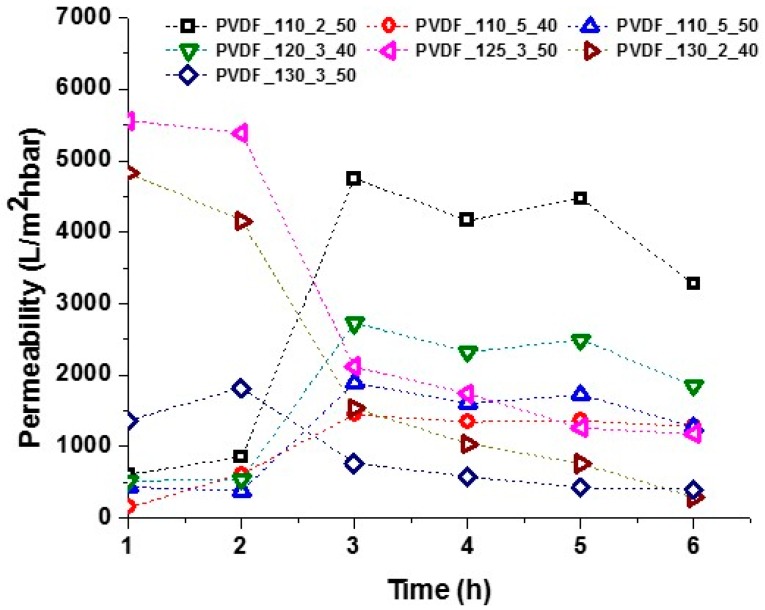
Separation of tap water using various laminated nanofibrous membranes.

The water filtration test was run to determine the flux of the membranes under tap water. Tap water is not pure, but contains several minerals, inorganics, hormones, fluorine compounds, etc. The aim of using water filtration is to compare the permeability of the membranes under various lamination conditions. The result of separation of tap water is given in Figure 12.

Membranes laminated at 125 °C showed that the permeability of the membranes increased sharply after two h of filtration, but then slightly decreased. The permeability of the membranes depends mainly on their hydrophilicity, thickness, pore size, and porosity. Herein, the membranes had more or less the same pore size and similar contact angles. It is well known that membrane permeability decrease over time due to membrane fouling and concentration polarisation [6,24,30].

In contrast, membranes laminated at higher than 125 °C showed that the permeability of the membranes was very high at the beginning, but then decreased drastically. There is a complex dependence between lamination temperature and membrane permeability.

During heat treatment at high temperature, recrystallisation of electrospun PVDF membrane occurred during heat treatment due to the polymer chains relaxing from disorganisation. As a result, there is a possibility of PVDF crystallites in different perfections. Double-melting peak were observed after annealing the melted PVDF samples at temperatures of 110–150 °C, which could be mainly ascribed to remelting and recrystallisation during annealing [42,43]. In another work, electrospun PVDF nanofibre layers were annealed at temperatures ranging from 40–100 °C. No visible changes were observed on SEM, while Raman analysis showed increases in both spectral intensity and beta to alpha phase ratio. The results indicated that thermal annealing had resulted in more β phase conversion, either at the expense of α phase or amorphous phase [44].

Results indicate that lamination temperature has an important role in membrane permeability. It was found that the membranes laminated below 125 °C were the best candidates for liquid filtration. The reason could be due to part recrystallisation of electrospun PVDF nanofibre membranes at high temperatures, which could change the membrane properties. Another reason is that below 125 °C membranes had higher air permeability compared to others, which could result in higher membrane porosity. After three h of filtration test, the permeability decreased very slightly for all the membranes. It is possible to say that the permeability behaviour of the membrane can be judged after three h of filtration test. In this case, PVDF_110_2_50 and PVDF_120_3_40 were promising membranes for liquid filtration.

## 4. Conclusions

The effects of laminating conditions (temperature, time, and force of lamination) on laminated nanofibrous membrane properties were investigated to increase the possible application of the membranes for both air and water filtration.

The surface morphology of the nanofibre web after lamination was investigated by using scanning electron microscopy, water contact angle, and average pore size in order to consider morphology changes. It was observed that the fibre diameter of the membranes remained unchanged under various lamination conditions. On the other hand, the water contact angle changed depending on the lamination conditions. It was found that lamination time played a major role in the pore size of the membranes. In addition, to compare breathability and the bursting strength of laminates, air permeability, and burst-pressure tests were run to select the best lamination conditions. It was found that all the membranes showed very low air permeability with high delamination resistance. Furthermore, air and water filtration tests were run to examine membrane performance and efficiency.

These results indicate that laminating temperature is an effective parameter for water permeability of the membranes. Thus, varying lamination parameters could lead to the development of membranes with different levels of air and water filtration efficiencies depending on need and use.

## Figures and Tables

**Table 1:** Tensile properties of the two selected membranes.

Sample	Tensile Strength (N/25 mm)		Elongation at Break (%)	
	MD	CD	MD	CD
PVDF_130_2_40	94.45	89.143	49.86	70.37
PVDF_130_3_50	94.32	87.37	52.52	68.38

## Appendix A

**Table A1:** Lamination condition and the abbreviation of the samples.

Nanofibre	Temperature (°C)	Time (min)	Force of Lamination (kN)	Abbreviation
PVDF	110	2	30	PVDF_110_2_30
	110	2	40	PVDF_110_2_40
	110	2	50	PVDF_110_2_50
	110	2	100	PVDF_110_2_100
	110	3	30	PVDF_110_3_30
	110	3	40	PVDF_110_3_40
	110	3	50	PVDF_110_3_50
	110	3	100	PVDF_110_3_100
	110	5	30	PVDF_110_5_30
	110	5	40	PVDF_110_5_40
	110	5	50	PVDF_110_5_50
	110	5	100	PVDF_110_5_100
	120	2	30	PVDF_120_2_30
	120	2	40	PVDF_120_2_40
	120	2	50	PVDF_120_2_50
	120	2	100	PVDF_120_2_100
	120	3	30	PVDF_120_3_30
	120	3	40	PVDF_120_3_40
	120	3	50	PVDF_120_3_50
	120	3	100	PVDF_120_3_100
	120	5	30	PVDF_120_5_30
	120	5	40	PVDF_120_5_40
	120	5	50	PVDF_120_5_50
	120	5	100	PVDF_120_5_100
	125	2	30	PVDF_125_2_30
	125	2	40	PVDF_125_2_40
	125	2	50	PVDF_125_2_50
	125	2	100	PVDF_125_2_100
	125	3	30	PVDF_125_3_30
	125	3	40	PVDF_125_3_40
	125	3	50	PVDF_125_3_50
	125	3	100	PVDF_125_3_100
	125	5	30	PVDF_125_5_30
	125	5	40	PVDF_125_5_40
	125	5	50	PVDF_125_5_50
	125	5	100	PVDF_125_5_100
	130	2	30	PVDF_130_2_30
	130	2	40	PVDF_130_2_40
	130	2	50	PVDF_130_2_50
	130	2	100	PVDF_130_2_100
	130	3	30	PVDF_130_3_30
	130	3	40	PVDF_130_3_40
	130	3	50	PVDF_130_3_50
	130	3	100	PVDF_130_3_100
	130	5	30	PVDF_130_5_30
	130	5	40	PVDF_130_5_40
	130	5	50	PVDF_130_5_50
	130	5	100	PVDF_130_5_100

**Table A2:** Air permeability and maximum burst pressure of the membranes.

Abbreviation	Air Permeability (Lm^−2^s^−1^)	Max. Burst Pressure (kPa)
PVDF_110_2_30	15.33 ± 2.04	138.00 ± 6.16
PVDF_110_2_40	15.10 ± 1.84	154.00 ± 14.17
* PVDF_110_2_50	12.49 ± 2.74	276.00 ± 15.78
PVDF_110_2_100	3.59 ± 0.63	213.00 ± 7.12
PVDF_110_3_30	14.40 ± 1.34	149.66 ± 8.81
PVDF_110_3_40	12.50 ± 1.71	162.66 ± 4.19
PVDF_110_3_50	11.73 ± 2.10	151.00 ± 20.61
PVDF_110_3_100	3.91 ± 1.29	184.00 ± 34.71
PVDF_110_5_30	9.92 ± 0.94	146.66 ± 15.17
*PVDF_110_5_40	9.52 ± 1.75	194.66 ± 13.37
*PVDF_110_5_50	8.74 ± 1.82	203.00 ± 16.19
PVDF_110_5_100	3.96 ± 0.55	345.33 ± 26.41
PVDF_120_2_30	10.51 ± 2.01	90.33 ± 10.34
PVDF_120_2_40	9.71 ± 2.04	117.66 ± 34.12
PVDF_120_2_50	7.64 ± 1.26	146.33 ± 7.93
PVDF_120_2_100	3.73 ± 0.38	201.66 ± 13.60
PVDF_120_3_30	9.74 ± 1.64	100.33 ± 31.33
* PVDF_120_3_40	9.15 ± 1.08	237.00 ± 10.61
PVDF_120_3_50	7.64 ± 0.97	116.00 ± 24.85
PVDF_120_3_100	4.16 ± 0.14	176.00 ± 15.25
PVDF_120_5_30	8.42 ± 0.66	84.00 ± 19.50
PVDF_120_5_40	8.70 ± 0.19	104.33 ± 57.56
PVDF_120_5_50	6.14 ± 0.97	116.00 ± 31.90
PVDF_120_5_100	3.57 ± 0.52	237.00 ± 34.92
PVDF_125_2_30	9.79 ± 1.16	93.00 ± 23.10
PVDF_125_2_40	6.98 ± 2.22	103.33 ± 17.80
PVDF_125_2_50	7.41 ± 2.53	114.66 ± 33.73
PVDF_125_2_100	5.61 ± 0.77	174.66 ± 6.02
PVDF_125_3_30	9.68 ± 1.23	103.00 ± 28.16
PVDF_125_3_40	8.38 ± 2.58	116.33 ± 15.76
*PVDF_125_3_50	8.48 ± 1.75	183.33 ± 14.29
PVDF_125_3_100	3.78 ± 0.38	178.33 ± 25.63
PVDF_125_5_30	8.12 ± 1.22	126.33 ± 33.21
PVDF_125_5_40	7.20 ± 1.28	135.33 ± 8.29
PVDF_125_5_50	5.76 ± 0.45	121.66 ± 9.03
PVDF_125_5_100	2.09 ± 0.14	225.33 ± 30.94
PVDF_130_2_30	10.65 ± 1.88	137.00 ± 15.63
* PVDF_130_2_40	9.25 ± 1.19	294.33 ± 16.05
PVDF_130_2_50	9.19 ± 0.71	108.33 ± 19.94
PVDF_130_2_100	4.53 ± 0.19	182.33 ± 26.66
PVDF_130_3_30	7.47 ± 0.92	141.66 ± 13.96
PVDF_130_3_40	6.95 ± 1.95	132.33 ± 12.73
* PVDF_130_3_50	7.16 ±0.89	197.00 ± 15.00
PVDF_130_3_100	3.34 ± 0.24	161.66 ± 29.49
PVDF_130_5_30	7.6 ± 0.70	104.00 ± 21.85
PVDF_130_5_40	4.13 ± 0.09	157.00 ± 20.08
PVDF_130_5_50	4.30 ± 0.62	203.66 ± 17.48
PVDF_130_5_100	3.81 ± 0.68	123.33 ± 23.54

* Selected membranes.
